# DARS2 overexpression is associated with PET/CT metabolic parameters and affects glycolytic activity in lung adenocarcinoma

**DOI:** 10.1186/s12967-023-04454-3

**Published:** 2023-08-26

**Authors:** Xu-Sheng Liu, Ling-Ling Yuan, Yan Gao, Xing Ming, Yao-Hua Zhang, Yu Zhang, Zi-Yue Liu, Yi Yang, Zhi-Jun Pei

**Affiliations:** 1 Department of Nuclear Medicine, Hubei Provincial Clinical Research Center for Umbilical Cord Blood Hematopoietic Stem Cells, Taihe Hospital, Hubei University of Medicine, Shiyan, 442000 China; 2 Department of Pathology, Taihe Hospital, Hubei University of Medicine, Shiyan, 442000 China; 3 Department of Infection Control, Taihe Hospital, Hubei University of Medicine, Shiyan, 442000 China

**Keywords:** Lung adenocarcinoma, SUVmax, PET/CT, DARS2, Glycolysis

## Abstract

**Background:**

This study investigated the correlation between the expression of DARS2 and metabolic parameters of ^18^F-FDG PET/CT, and explored the potential mechanisms of DARS2 affecting the proliferation and glycolysis of lung adenocarcinoma (LUAD) cells.

**Methods:**

This study used genomics and proteomics to analyze the difference in DARS2 expression between LUAD samples and control samples. An analysis of 62 patients with LUAD who underwent ^18^F-FDG PET/CT examinations before surgery was conducted retrospectively. The correlation between DARS2 expression and PET/CT metabolic parameters, including SUVmax, SUVmean, MTV, and TLG, was examined by Spearman correlation analysis. In addition, the molecular mechanism of interfering with DARS2 expression in inhibiting LUAD cell proliferation and glycolysis was analyzed through in vitro cell experiments.

**Results:**

DARS2 expression was significantly higher in LUAD samples than in control samples (p < 0.001). DARS2 has high specificity (98.4%) and sensitivity (95.2%) in the diagnosis of LUAD. DARS2 expression was positively correlated with SUVmax, SUVmean, and TLG (p < 0.001). At the same time, the sensitivity and specificity of SUVmax in predicting DARS2 overexpression in LUAD were 88.9% and 65.9%, respectively. In vitro cell experiments have shown that interfering with DARS2 expression can inhibit the proliferation and migration of LUAD cells, promote cell apoptosis, and inhibit the glycolytic activity of tumor cells by inhibiting the expression of glycolytic related genes SLC2A1, GPI, ALDOA, and PGAM1.

**Conclusions:**

Overexpression of DARS2 is associated with metabolic parameters on ^18^F-FDG PET/CT, which can improve LUAD diagnosis accuracy. DARS2 may be a useful biomarker to diagnose, prognosis, and target treatment of LUAD patients.

**Supplementary Information:**

The online version contains supplementary material available at 10.1186/s12967-023-04454-3.

## Introduction

Lung cancer, as one of the most common malignant tumors in the world, is a serious threat to public health [1]. Lung cancer is generally divided into non-small cell lung cancer (NSCLC) and small cell lung cancer (SCLC), accounting for 85% and 15% of the total population of lung cancer respectively. In NSCLC, lung adenocarcinoma (LUAD) is the most common pathological type [2–4]. The treatment of lung cancer is mainly surgery, chemotherapy or a combination of the two, but the prognosis is poor, and the recurrence rate of advanced lung cancer is high [2, 5, 6]. The occurrence of LUAD is related to many factors. Consequently, it holds significant importance to delve deeper into the molecular mechanism of LUAD, discover novel methods for early detection, and develop more efficient treatment strategies. This can lead to enhanced prognostic outcomes for patients with lung cancer and contribute to the worldwide efforts to alleviate the burden of this malignancy.

DARS2 (Aspartyl-TRNA Synthetase 2, Mitochondrial) is a Protein Coding gene. The protein encoded by this gene belongs to the class-II aminoacyl-tRNA synthetase family. It is a mitochondrial enzyme responsible for the first step of mitochondrial protein synthesis [7, 8]. The earliest study found that DARS2 gene is mainly related to a white matter encephalopathy characterized by brain stem and spinal cord involvement with elevated lactic acid [7, 9]. Some studies have shown that the expression of DARS2 has a certain predictive effect on bladder cancer (BLCA) and LUAD [10–15], and has been identified as an hepatocarcinogenesis (HCC) oncogene that promotes the progression of HCC cell cycle and inhibits apoptosis of HCC cells [16]. In previous studies [17], we found that LUAD cell proliferation is inhibited by interfering with the expression of DARS2. However, the mechanism by which DARS2 affects the development of LUAD is unclear.

The aerobic glycolysis of tumor cells, namely Warburg effect, is one of the important ways of tumor development [18]. ^18^F-fluorodeoxyglucose (^18^F-FDG) Positron emission tomography/computed tomography (PET/CT) scan is a non-invasive imaging method, which is widely used in the diagnosis, staging and prognosis evaluation of diseases [19, 20]. FDG is a glucose analogue, which cannot be further metabolized after being transported into cells through glucose transport enzyme, but can only be gathered in cells and then captured by PET. Therefore, the glycolysis ability of tumor cells can be reflected by PET/CT metabolic parameters [21, 22]. Metabolic parameters include maximum standardized uptake value (SUVmax), mean standardized uptake value (SUVmean), total lesion glycolysis (TLG) and metabolic tumor volume (MTV). SUVmax represents the maximum standardized uptake value, which indicates the highest level of glucose uptake in a tumor region. SUVmean is the average standardized uptake value within the tumor, providing a measure of overall glucose metabolism. TLG is the product of SUV mean and tumor volume and represents the total glucose consumption within the tumor. Lastly, MTV is the volume of the tumor with elevated glycolytic activity, determined by a threshold value of SUV [19, 23].

By utilizing bioinformatics techniques, we examined the expression levels of DARS2 in different LUAD datasets and investigated its prognostic value for individuals with LUAD. Interfering with DARS2 expression in LUAD cells was used to investigate DARS2’s effects on proliferation and migration. At the same time, we retrospectively analyzed the correlation between DARS2 expression and PET/CT metabolic parameters in tumor sections of 62 patients with LUAD to analyze the potential relationship between DARS2 and glycolysis of LUAD. In addition, cell and molecular experiments confirmed the effect of DARS2 expression on aerobic glycolysis in LUAD cells. This study seeks to identify find a new molecular target with the function of early diagnosis and treatment, and to provide a basis for the subsequent development of new imaging targets.

## Materials and methods

### Bioinformatics analysis of DARS2 in LUAD

We downloaded the mRNA microarray dataset from TCGA (https://tcga-data.nci.nih.gov/tcga/) [24] and GEO (http://www.ncbi.nlm.nih.gov/geo) [25]. The TCGA LUAD dataset includes 59 normal samples and 535 LUAD samples, among which the LUAD sample population includes Asian, black or African Americans, and white people. GSE19188 includes 91 NSCLC and 65 normal lung specimens, and 45 of 91 NSCLC specimens are LUAD. GSE30219 includes 293 lung cancer and 14 normal lung specimens, and 85 of 293 lung cancer specimens are LUAD. Limma package was used for differential gene analysis of TCGA LUAD, GSE19188 and GSE30219 datasets [26]. The threshold was set to p.adj < 0.05 and | log_2_ FC (fold-change) |> 0.5. We used Wilcoxon rank sum test to analyze the difference of DARS2 expression between normal samples and LUAD samples in the above three datasets. We further analyzed the clinical sample information of TCGA LUAD dataset, and studied the relationship between the expression of DARS2 and pathological stage and patient prognosis. Finally, we analyzed the TCGA LUAD dataset to analyze the potential relationship between DARS2 expression and glycolysis related genes.

### Patient samples

This study reviewed 62 LUAD patients who were pathologically confirmed and surgically removed in Taihe Hospital from August 2018 to June 2020. These patients received ^18^F-FDG PET/CT imaging before surgery, and did not receive any radiotherapy or chemotherapy before imaging. These patients have complete and available clinical pathological data. The T stages refer to the extent of the primary tumor, the N stages refer to the involvement of regional lymph nodes, and TNM is an abbreviation for a staging system used in cancer, which includes T, N, and M (metastasis) stages.

### Immunohistochemical (IHC) assay

IHC staining was performed with reference to previous research methods [27]. The tumors of the above 62 patients and their paired adjacent tissues were embedded in paraffin and sectioned. Incubate with DARS2 antibody (13807-1-AP, Proteintech, China, 1:200) and corresponding secondary antibody (ab6802, abcam, USA, 1:500). Two professional pathologists independently analyzed the IHC data. The scoring standard is 0 (negative), 1 (weakly positive), 2 (moderately positive) and 3 (strongly positive). 0–1 scores were defined as low expression and 2–3 scores as high expression.

### Cell culture and treatment

The human LUAD cell line H1975 was purchased from BeNa Culture Collection (BNCC340345, BNCC, China). Cell culture the media used included RPMI-1640 complete medium (KGM31800S, KeyGEN, China) and RPMI-1640 incomplete medium (KGM31800N, KeyGEN, China). Following the manufacturer’s instructions, the cells were transfected with DARS2 siRNA using Lipofectamine 3000 transfection reagent (L3000015, Invitrogen, USA). Detailed siRNA sequences are shown in Additional file 1: Table S1.

### Total RNA extraction and quantitative real-time PCR

Total RNA was extracted from LUAD cell lines using Trizon Reagent (CW0580S, CWBIO, China). The cDNA was synthesized using HiScript II Q RT SuperMix for qPCR (R223-01, Vazyme, China). qRT-PCR was performed using Real-Time PCR System (CFX Connect™, Bio-Rad, USA) and 2−ΔΔCt method was used to quantify the relative expression levels. We used β-actin as an internal control for normalization. Detailed primer sequences are shown in Additional file 2: Table S2. The selection of glycolytic related genes is based on previous studies [28], mainly including SLC2A1, HK2, GPI, PFKL, ALDOA, GAPDH, PGK1, PGAM1, ENO1, PKM2 and LDHA.

### Western blot

Treated LUAD cells were extracted by RIPA lysis buffer (C1053, Beijing Pulilai Gene Technology Co., Ltd., China). The protein concentrations were measured by BCA method (E-BC-K318-M, Elabscience, China). 40 μg of protein was separated by 10% SDS-polyacrylamide gels. The protein samples were transferred onto PVDF membranes (IPVH00010, Millipore, USA). The membranes were blocked with 5% BSA for 2 h. Followed by incubation with primary antibodies, including Rabbit Anti-DARS2 (DF12593, Affinity, China, 1/1000) and Mouse Anti-β-Actin (HC201, TransGen Biotech, China, 1/2000). The membranes were washed with TBST, and then incubated with corresponding secondary antibodies, including HRP conjugated Goat Anti-Mouse IgG (H+L) (GB23301, Servicebio, China, 1/2000) and HRP conjugated Goat Anti-Rabbit IgG (H+L) (GB23303, Servicebio, China, 1/2000), at room temperature for 1 h. Finally, the proteins were visualized by an enhanced chemiluminescence (ECL) detection kit (RJ239676, Thermo Fisher Scientific, Inc., USA). We captured the chemiluminescent signal of protein bands using the Tanon-5200 chemiluminescence imaging system (Tanon, Shanghai, China). The optical density of protein bands was quantified by using ImageJ software (Image J Software Inc., USA).

### EdU proliferation assay

The EdU kit (C0078S, Beyotime, China) was used to detect the proliferative activity of LUAD cells transfected with siRNA. For a detailed experimental protocol, please refer to our previous research [29].

### CCK-8 assay for cellular viability

After transfection of siRNA into LUAD cells for 48 h, the transfected cells were seeded into and cultured in 96 well plates (5 × 10^3^ cells/well). Add 10 μL of CCK-8 solution (KGA317, KeyGen, China) to each well at 24, 48, and 72 h of incubation, respectively. After 2 h, the culture was suspended, and the optical density at 450 nm per well was measured using a microplate reader (SuPerMax3100, Shanghai Flash spectrum biological technology co., China).

### Wound healing assay

Seed transfected cells into a six-well culture plate until they reach 90% confluence. Use the tip of a 200  μL pipette to scratch the cells. Observe cell migration at 0, 24, and 48 h under a microscope. Use Image J software to quantify the scratched area.

### Matrigel invasion assay

Cell culture medium was removed from the cell plate, and cells were fixed for 15 min. Afterwards, PBS was added for a 5-min wash, followed by the addition of a prepared 0.1% crystal violet solution for 1 h of staining. After staining, the inner chamber cells were wiped off with a cotton swab, and the chamber was inverted onto a glass slide for imaging using a microscope (BX43, OLYMPUS, Japan). Finally, cell counting was performed using ImageJ software.

### Apoptotic assay

Annexin V-FITC/PI Cell Apoptosis Detection Kit (AP101-100 kit, MULTI SCIENCES, China) is used to detect cell apoptosis. Through NovoCyte™ Flow cytometry (NovoCell 2060R, ACEA Biosciences Inc., USA) detects apoptosis at a wavelength of 488 nm [30].

### PET/CT imaging and data analysis

PET/CT imaging of ^18^F-FDG was used to assess glucose metabolism. Referring to previous studies [27, 29], patients fasted for 6–8 h before intravenous injection of ^18^F-FDG (3.7–4.1 MBq/kg). After 50 min of drug injection, PET/CT imaging was performed with Biograph mCT 64 system (Siemens Healthcare, Germany). Experienced nuclear medicine doctors draw the region of interest (ROI) around the tumor, and automatically calculate and record the maximum and mean standardized uptake value (SUVmax and SUVmean), total lesion glycolysis (TLG) and metabolic tumor volume (MTV) of each ROI through the system.

### Gene set enrichment analysis (GSEA) analysis

In order to further analyze whether DARS2 participates in the glycolysis process of LUAD, we used clusterProfiler package [31] to conduct GSEA analysis of DARS2 differential genes in TCGA LUAD dataset. According to the expression of DARS2, the TCGA LUAD dataset was divided into high and low groups. The differential genes of the two groups were analyzed using DESeq2 package [32], and the gene that can encode the protein was selected as the final differential gene for GSEA analysis. The reference genes were h.all.v7.2.symbols.gmt [Hallmarks] and c2.cp.v7.2.symbols.gmt [Curated]. Data with the FDR (q-value) < 0. 25 and p < 0. 05 were considered statistically significant. The gene set database refers to MSigDB Collections (https://www.gsea-msigdb.org/gsea/msigdb/collections.jsp#C2).

### 2-NBDG uptake assay

Cells were seeded in 96 well plates (2 × 10^4^ cells/well). Twenty-four hours after transfection of siRNA, cells were washed with PBS. Incubate cells with 50 μM 2-NBDG (HY-116215, MCE, USA) in glucose-free DMEM for 30 min (37 °C, 5% CO_2_). A three-time wash with warm PBS was performed after the incubation period. The mean fluorescence intensity (MFI) of 2-NBDG was analysed using NovoCyte™ Flow cytometry (NovoCyte 2060R, ACEA Bioscience, Inc., USA) [33]. 2-NBDG is a fluorescently labelled glucose-like compound that enters cells by competing with the glucose transporter protein GLUT for binding. Within the cell, 2-NBDG is enzymatically hydrolysed, releasing fluorescence as a measurement indicator.

### Measurement of lactate

The Lactate Colorimetric Assay Kit (E-BC-K044-M, Elabscience) was used to measure lactate levels in the culture medium. According to the manufacturer's instructions, we measured the absorbance at 530 nm [33].

### Statistics analysis

The statistical analyses and plotting were performed using the Xiantao online database tool (https://www.xiantaozi.com/) and GraphPad Prism statistical program. Xiantao is a powerful data analysis tool designed specifically for biomedical research, primarily used for bioinformatics analysis. It supports common statistical tests such as t-tests, analysis of variance, and regression analysis, making it applicable to a wide range of experimental designs. Additionally, Xiantao provides advanced data visualization capabilities, enabling researchers to generate high-quality charts. The measurement data results are expressed as mean ± standard deviation. Two-tailed p values < 0.05 were considered statistically significant. ns means not significant; *p < 0.05; **p < 0.01; ***p < 0.001; ****p < 0.0001.

## Result

### DARS2 overexpression in LUAD

**Fig. 1:**
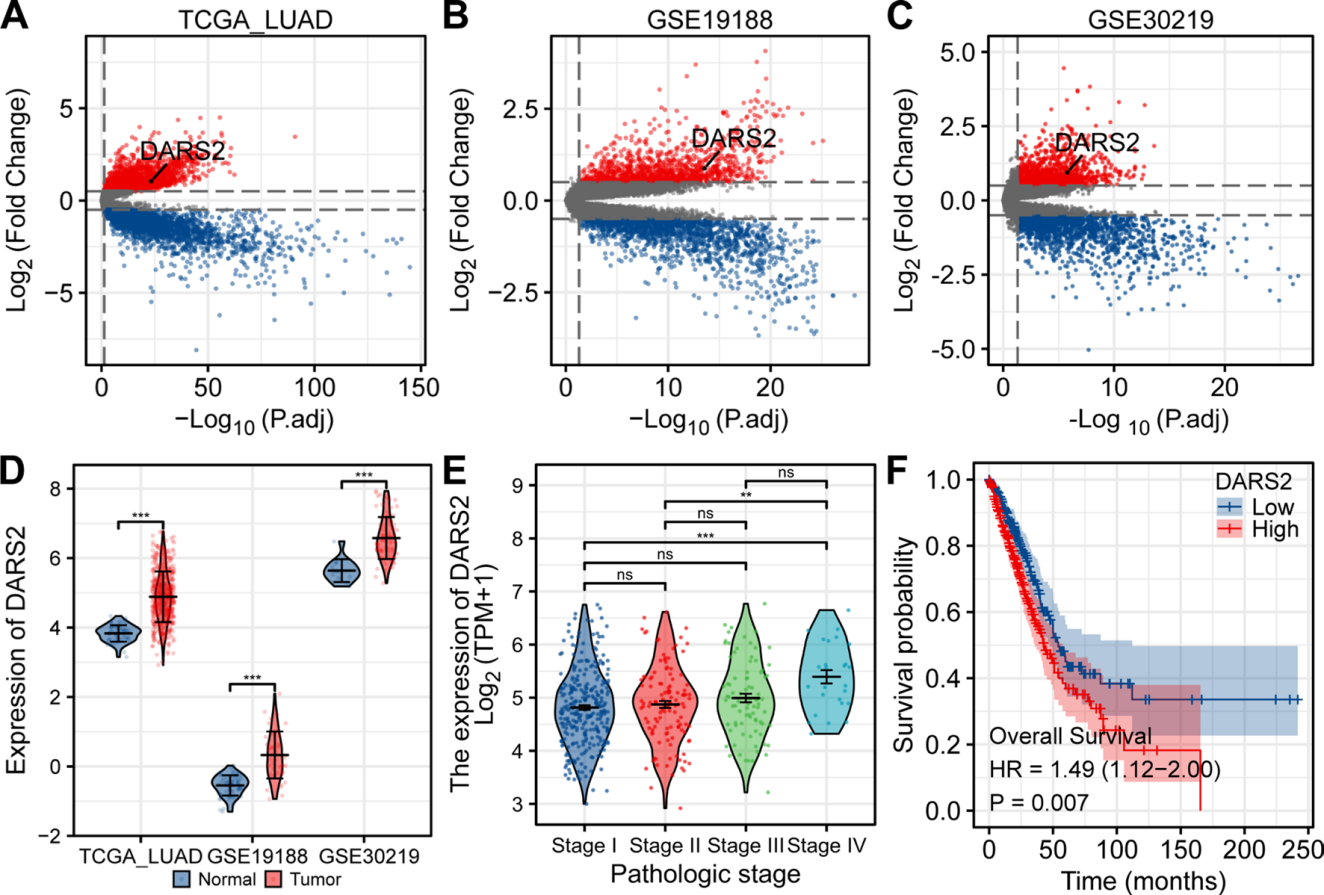
DARS2 overexpression in lung adenocarcinoma and its correlation with clinicopathological features. **A**–**C** The volcano map shows the limma analysis results of TCGA LUAD, GSE19188 and GSE30219 datasets. **D** There was a significant increase in DARS2 expression in the tumor group compared to the normal group, in TCGA LUAD, GSE19188 and GSE30219 datasets. **E** The expression difference of DARS2 in different pathological stages in TCGA LAUD dataset. **F** Kaplan–Meier curve shows the overall survival of TCGA LUAD datasets. ns means not significant; **p < 0.01, ***p < 0.001

**Fig. 2:**
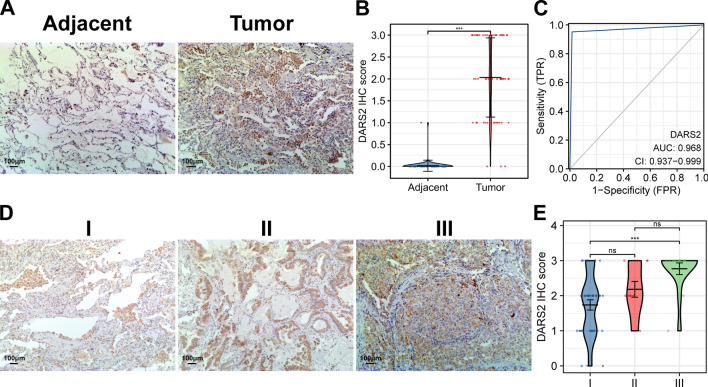
Immunohistochemistry staining of lung adenocarcinoma samples. **A**, **B** Representative DARS2 IHC images and scores in adjacent normal tissue and LUAD tissue (adjacent = 62, tumor = 62). **C** DARS2’s diagnostic value in LUAD patients shown in the ROC curve. **D**, **E** Representative IHC images and DARS2 scores in different pathological stages of LUAD (I = 38, II = 11, III = 13). ns means not significant; ***p < 0.001

Limma analysis results showed that in the TCGA LUAD dataset, 3209 genes were up-regulated and 3178 were down-regulated, of which DARS2 (log_2_ FC = 1.049, p.adj = 4.55E−24) was significantly up-regulated (Fig. 1A). In GSE19188 dataset, 1235 genes were up-regulated and 1678 were down regulated, among which DARS2 (log_2_ FC = 0.875, p.adj = 2.79E−14) was significantly up-regulated (Fig. 1B). In GSE30219 dataset, 1526 genes were up-regulated and 1735 were down regulated, among which DARS2 (log_2_ FC = 0.937, p.adj = 1.67E−06) was significantly up-regulated (Fig. 1C). Wilcoxon rank sum test results showed that in TCGA LUAD, GSE19188 and GSE30219 datasets, there was a significant increase in DARS2 expression in the tumor group compared to the normal group (Fig. 1D, p < 0.001). The IHC staining results showed that the expression of DARS2 in LUAD patients was significantly higher than that in the adjacent tissues (Fig. 2A, B, p < 0.001). The ROC curve analyses demonstrated that DARS2 displayed superior diagnostic accuracy (AUC = 0.968, 95% CI 0.937–0.999) which could distinguish the LUAD patients from normal controls (Fig. 2C). DARS2 has high specificity (98.4%) and sensitivity (95.2%) in the diagnosis of LUAD.

### Association between DARS2 expression and clinicopathological characteristics of lung adenocarcinoma patients

Bioinformatics found that the expression level of DARS2 in the stage IV LUAD was significantly higher than that in the stage I and stage II (Fig. 1E, p < 0.001). Kaplan–Meier analysis demonstrated that patients with high DARS2 expression had poorer prognosis (Fig. 1F, p = 0.007). The analysis results also showed that in different pathological stages, the IHC score of DARS2 was significantly higher in stage III samples than that in stage I samples (Fig. 2D, E, p < 0.001). The 62 LUAD patients were divided into DARS2 low expression group (n = 18) and high expression group (n = 44). Subsequent analysis showed that DARS2 expression was significantly correlated with differentiation (p = 0.010), N stage (p = 0.007) and TNM stage (p = 0.023), but not with gender, age and T stage (p > 0.05) (Table 1).

**Table 1:** Relationship between DARS2 expression and patients features

Characteristic	DARS2^Low^	DARS2^High^	p
n	18	44	
Gender, n (%)			0.593
Female	11 (17.7%)	30 (48.4%)	
Male	7 (11.3%)	14 (22.6%)	
Age (years), n (%)			0.517
< 60	13 (21%)	28 (45.2%)	
≥ 60	5 (8.1%)	16 (25.8%)	
Differential, n (%)			0.010
High/moderately	16 (25.8%)	24 (38.7%)	
Poorly	2 (3.2%)	20 (32.3%)	
T stage, n (%)			0.167
T1	16 (25.8%)	32 (51.6%)	
T2–T4	2 (3.2%)	12 (19.4%)	
N stage, n (%)			0.007
N0	16 (25.8%)	23 (37.1%)	
N1–N3	2 (3.2%)	21 (33.9%)	
TNM stage, n (%)			0.023
I	15 (24.2%)	23 (37.1%)	
I–III	3 (4.8%)	21 (33.9%)	

### Interference with DARS2 expression inhibits proliferation and migration of LUAD cells

**Fig. 3:**
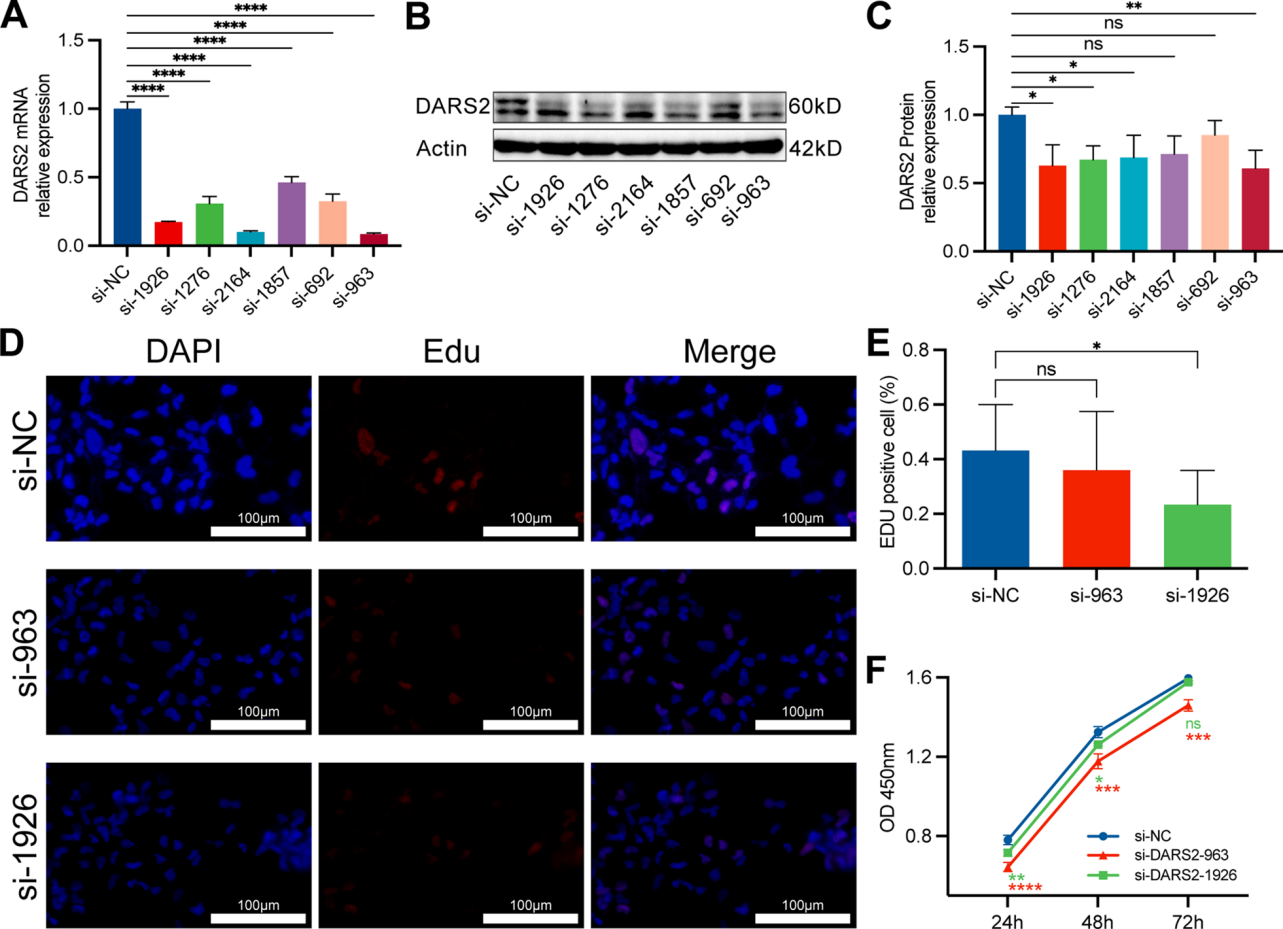
Interference DARS2 expression inhibited the proliferation of LUAD cells. **A** qRT-PCR verified the interference efficiency of 6 different sequence siRNAs. **B**, **C** WB verified the interference efficiency of 6 different sequences of siRNA. **D** Representative images of EdU staining. **E** Quantitative analysis of EdU staining. **F** CCK-8 assays were performed to evaluate the LUAD cell’s viability and growth. ns means not significant; *p < 0.05; **p < 0.01; ***p < 0.001; ****p < 0.0001

**Fig. 4:**
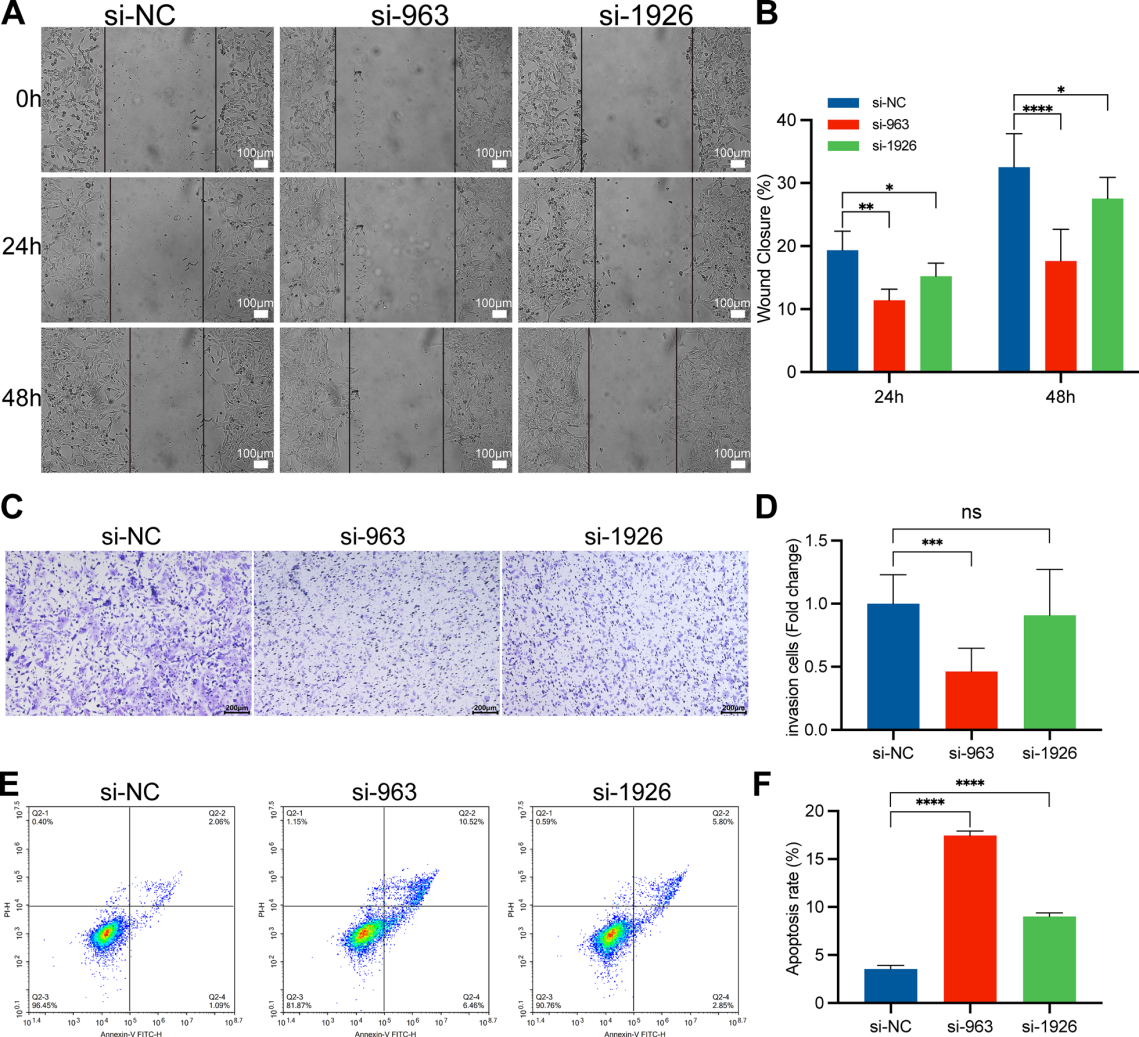
Interference with DARS2 expression affects the migration and apoptosis of LUAD cells. **A**, **B** Representative images and quantitative analysis of wound healing measurements in LUAD cells. **C**, **D** Representative images and quantitative analysis of cell invasion experiments in LUAD cells. **E** Representative pictures of apoptosis rate measured by flow cytometry. **F** Quantitative analysis of apoptosis. *p < 0.05; **p < 0.01; ***p < 0.001; ****p < 0.0001

To explore the inhibitory effect of interfering with DARS2 expression on LUAD cells, we constructed 6 siRNAs with different sequences to interfere with DARS2 expression. qRT-PCR and WB experiments verified the interference efficiency of these 6 siRNAs (Fig. 3A–C). Finally, we chose siRNA-963 (si-963) and siRNA-1926 (si-1926) for subsequent experiments. We used EdU proliferation assay and CCK-8 assay to measure cell viability. In EdU experiments, the si-1926 group’s cell viability significantly decreased compared to the control group (Fig. 3D, E, p < 0.05). Although there was no statistical difference between the si-963 group and the control group, there was also a downward trend. As shown in Fig. 3F, both siRNA groups had significantly lower cell viability than the control group in the CCK-8 experiment. In addition, wound healing measurements showed that interfering with the expression of DARS2 significantly inhibited the rate of wound healing (Fig. 4A, B). The Matrigel invasion assay revealed that the suppression of DARS2 expression significantly inhibited the invasive capability of LUAD cells (Fig. 4C, D). At the same time, flow cytometry results showed that the number of apoptotic cells in both siRNA groups increased significantly (Fig. 4E, F). It was further confirmed that suppression of DARS2 expression can significantly impede the proliferation of LUAD cells.

### Relationship between DARS2 IHC score and PET/CT metabolic parameters

**Fig. 5:**
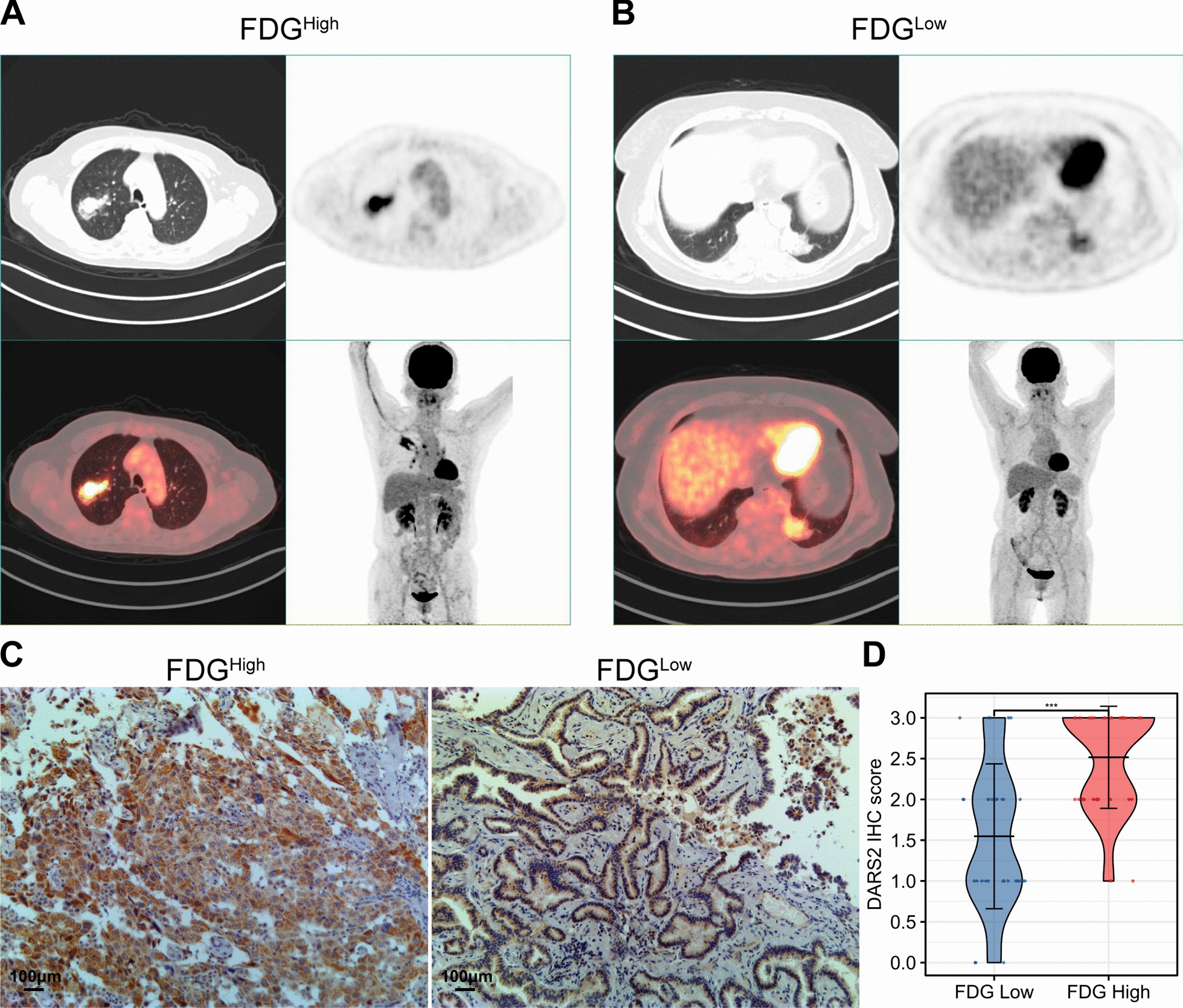
In patients with low or high ^18^F-FDG levels of LAUD, expression of DARS2 was compared. **A**, **B** Representative PET/CT images for LUAD patients with different SUVmax values. **C**, **D** IHC staining for DARS2 in LUAD patients with high (n = 31) and low (n = 31) SUVmax values. ***p < 0.001

**Fig. 6:**
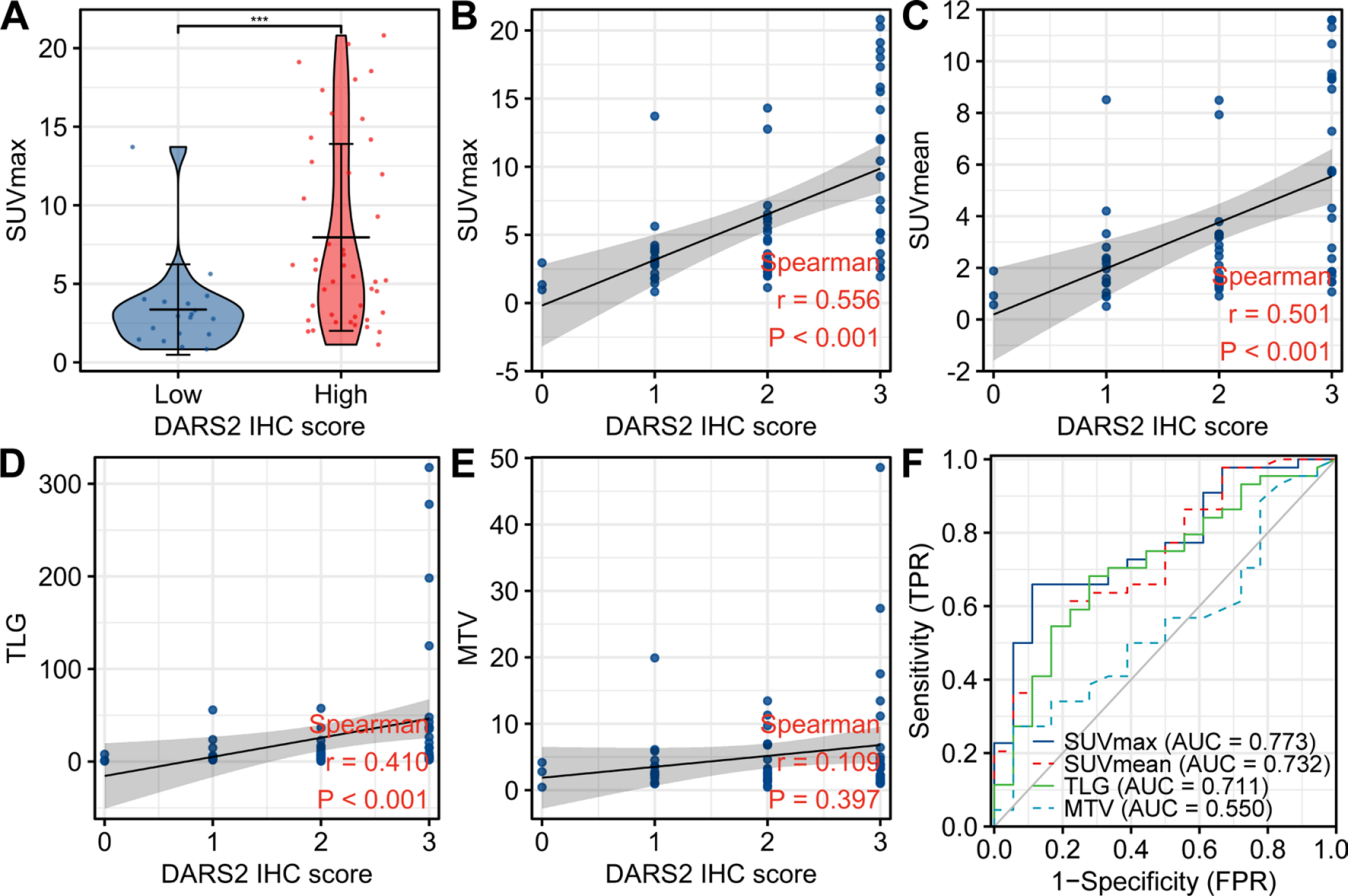
Correlation between DARS2 and PET/CT metabolic parameters in LUAD patient. **A** The difference of SUVmax between DARS2 high and low expression groups. A Scatter plots showing the correlation of SUVmax (**B**), SUVmean (**C**), TLG (**D**) and MTV (**E**) with the DARS2 IHC score. **F** ROC curve was used to analyze the ability of PET/CT metabolic parameters to predict the high and low outcomes of DARS2 expression. ***p < 0.001

By comparing PET metabolic parameter values and DARS2 IHC scores of LUAD patients, we found that high and low DARS2 IHC scores were statistically different from high and low SUVmax (p < 0.001), SUVmean (p = 0.005) and TLG (p = 0.025), but not from high and low MTV (p = 1) (Table 2). Figure 5A, B shows typical PET/CT images of LUAD patients with high and low SUVmax. The expression of DARS2 in patients with high SUVmax was significantly higher than that in patients with low SUVmax (Fig. 5C, D). Additionally, we found that the SUVmax value of patients in the high DARS2 expression group was significantly higher compared with the low DARS2 expression group (Fig. 6A). In order to further study the relationship between DARS2 expression and PET metabolic parameters, we conducted Spearman correlation analysis on the data. Results As shown in Fig. 6B–E, DARS2 protein level was significantly correlated with SUVmax (r = 0.556, p < 0.001), SUVmean (r = 0.501, p < 0.001) and TLG (r = 0.410, p < 0.001). However, there was no statistical correlation between DARS2 IHC score and MTV (r = 0.109, p = 0.397).

**Table 2:** Relationship between DARS2 expression and metabolic parameters

Characteristic	DARS2^Low^	DARS2^High^	p
n	18	44	
SUVmax, n (%)			<0.001
High	2 (3.2%)	29 (46.8%)	
Low	16 (25.8%)	15 (24.2%)	
SUVmean, n (%)			0.005
High	4 (6.5%)	27 (43.5%)	
Low	14 (22.6%)	17 (27.4%)	
TLG, n (%)			0.025
High	5 (8.1%)	26 (41.9%)	
Low	13 (21%)	18 (29%)	
MTV, n (%)			1.000
High	9 (14.5%)	22 (35.5%)	
Low	9 (14.5%)	22 (35.5%)

### SUVmax is the main predictor of DARS2 expression

In terms of predicting the high and low outcomes of DARS2 expression, ROC curve analysis showed that the prediction ability of SUVmax (AUC = 0.773, 95% CI 0.649–0.896), SUVmean (AUC = 0.732, 95% CI 0.595–0.868) and TLG (AUC = 0.711, 95% CI 0.571–0.852) had certain accuracy, while the prediction ability of MTV (AUC = 0.550, 95% CI 0.394–0.706) had low accuracy (Fig. 6F). The sensitivity and specificity of SUVmax in predicting DARS2 overexpression in LUAD were 88.9% and 65.9%, respectively.

### GSEA analysis of DARS2

**Fig. 7:**
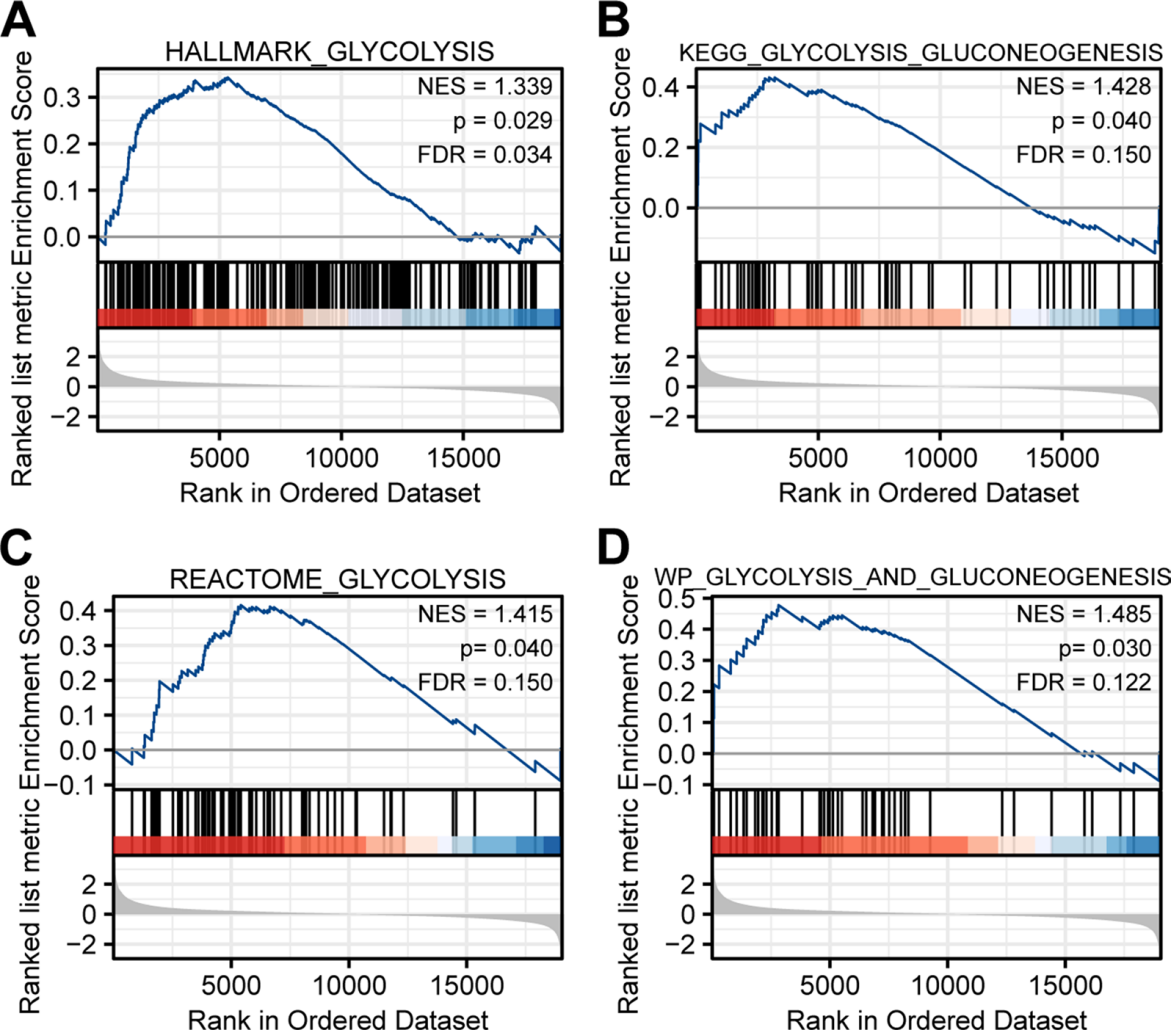
Gene set enrichment analysis (GSEA) analysis. **A**–**D** GSEA showed that the high expression of DARS2 was positively correlated with the pathway related to glycolysis

Through GSEA analysis (Fig. 7), we found that DARS2 differential genes were involved in glycolysis related pathways, including HALLMARK_GLYCOLYSIS (NES = 1.339, p = 0.029), KEGG_GLYCOLYSIS_GLUCONEOGENESIS (NES = 1.428, p = 0.040), REACTOME_GLYCOLYSIS (NES = 1.415, p = 0.040) and WP_GLYCOLYSIS_AND_GLUCONEOGENESIS (NES = 1.485, p = 0.030).

### Correlations between DARS2 expression and glycolysis in LUAD

**Fig. 8:**
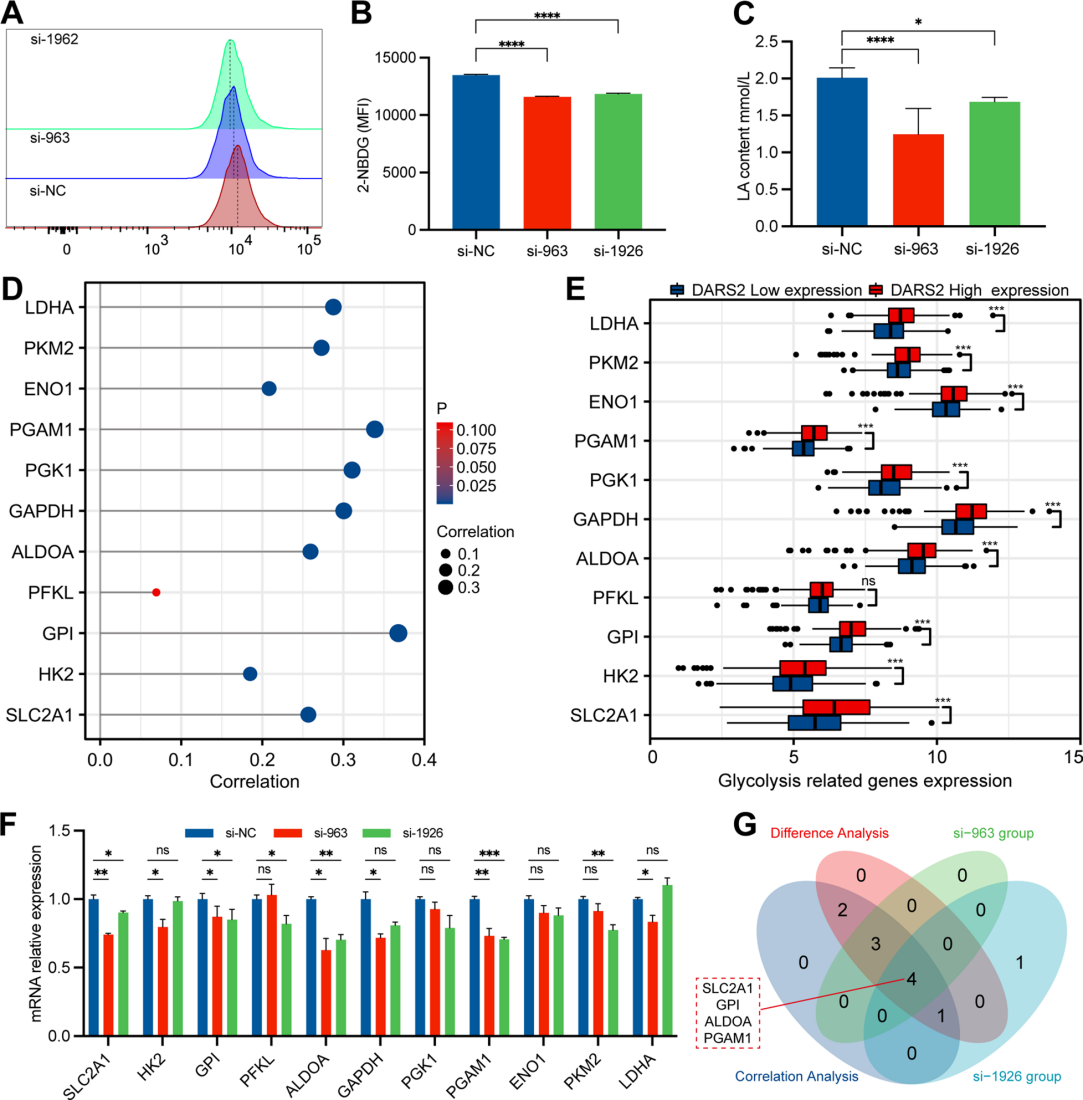
Correlations between DARS2 expression and glycolysis in LUAD. **A** Flow cytometry results of glucose uptake in LUAD cells determined by 2-NBDG staining. **B** Glucose uptake capacity was calculated using average fluorescence intensity. **C** Determination of lactate production in different groups of LUAD cells. **D** The lollipop diagram shows the correlation between DARS2 and the expression of glycolysis related genes. **E** Differential expression analysis of glycolysis related genes between DARS2 high and low expression groups. **F** Determination of the expression level of 11 glycolytic genes in different groups of LUAD cells. **G** The Venn diagram shows the positive genes that match the above three studies. ns means not significant; *p < 0.05; **p < 0.01; ***p < 0.001, ****p < 0.0001

**Fig. 9:**
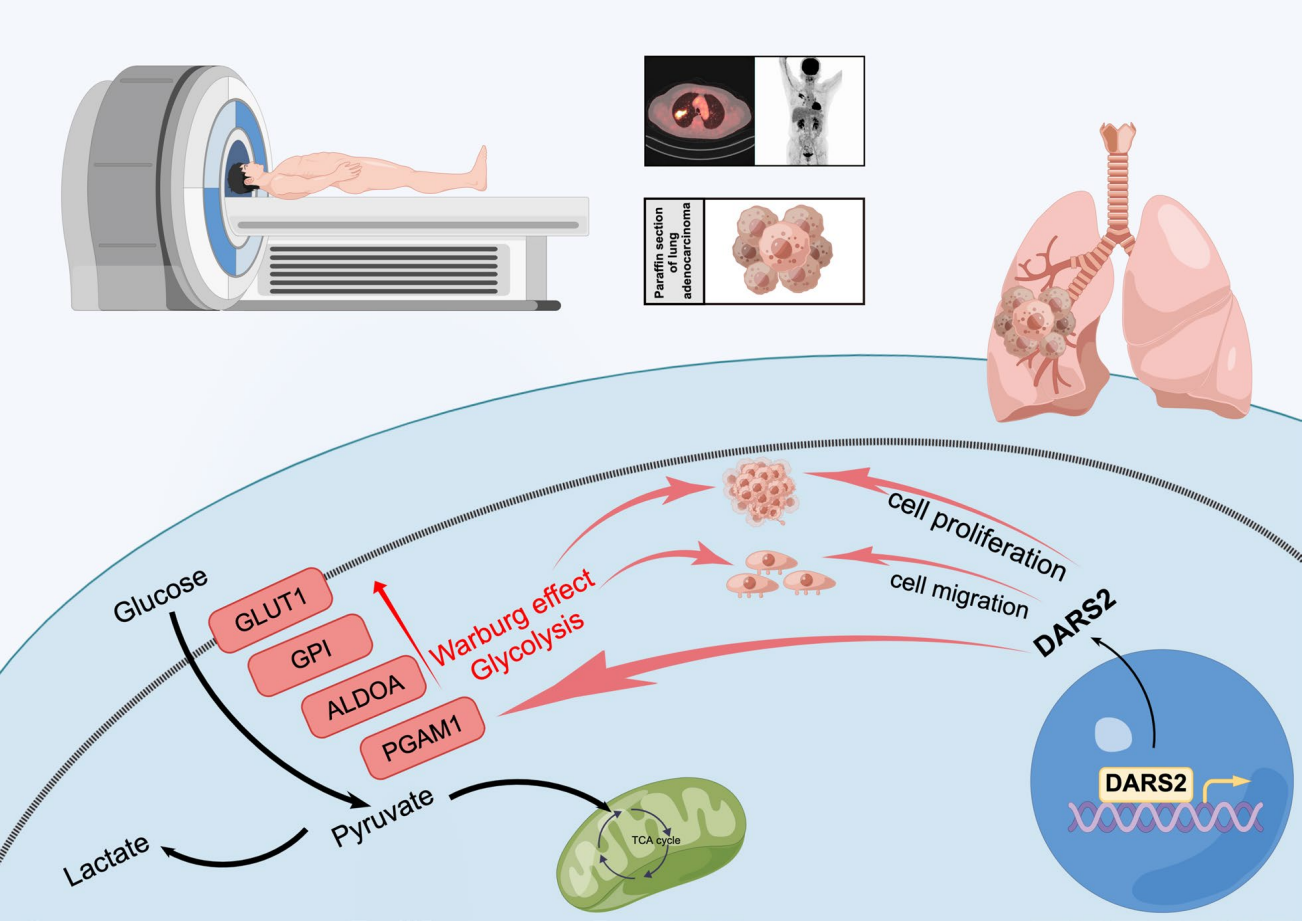
The research process and mechanism are shown in the figure. The figure was created by Figdraw (http://www.figdraw.com)

In order to further analyze the effect of DARS2 expression on the glycolysis of LUAD, we conducted glucose uptake experiments and lactate production experiments. The results of 2-NBDG fluorescence staining on flow cytometry showed that decrease in DARS2 expression significantly reduced 2-NBDG uptake in LUAD cells (Fig. 8A, B). The increase in lactate production is also a characteristic of the Warburg effect. The experimental results showed that the lactate production of LUAD cells in both siRNA groups decreased significantly compared to the control group (Fig. 8C). To clarify the potential mechanism of DARS2 affecting glycolysis in LUAD cells, we found that the expression of DARS2 was significantly positively correlated with several glycolysis related genes, including SLC2A1 (r = 0.257, p = 1.911E−09), HK2 (r = 0.185, p = 1.714E−05), GPI (r = 0.368, p = 1.579E−20), ALDOA (r = 0.259, p = 1.286E−09), GAPDH (r = 0.3, p = 1.627E−12), PGK1 (r = 0.311, p = 2.558E−13), PGAM1 (r = 0.339, p = 9.819E−16), ENO1 (r = 0.208, p = 1.225E−06), PKM2 (r = 0.273, p = 1.603E−10) and LDHA (r = 0.288, p = 1.461E−11), by analyzing the TCGA LUAD dataset (Fig. 8D). At the same time, according to the expression of DARS2, we also found that the expression level of SLC2A1, HK2, GPI, ALDOA, GAPDH, PGK1, PGAM1, ENO1, PKM2 and LDHA, in the high expression group was significantly higher than that in the low expression group (Fig. 8E, p < 0.001). At the same time, we detected the expression trend of these 11 glycolytic genes after transfection with siRNA through qRT-PCR experiment. The results showed that the expression of SLC2A1, GPI, ALDOA, and PGAM1 genes in both siRNA groups (si-963 and si-1962) decreased significantly compared to the control group (Fig. 8F, p < 0.05). However, HK2, PFKL, GAPDH, PKM2 and LDHA genes showed a significant downward trend in only one siRNA group (si-963/si-1962, Fig. 8F, p < 0.05). The expression of PGK1 and ENO1 genes did not significantly decrease compared to the control group (Fig. 8F, p > 0.05). The Venn diagram shows the positive genes that match the above three studies, namely SLC2A1, GPI, ALDOA and PGAM1 (Fig. 8G). The research process and mechanism are shown in Fig. 9.

## Discussion

LUAD is the most common subtype of lung cancer. The clinical symptoms of early LUAD are not obvious, and most of the symptomatic LUAD patients are in the late stage. These patients have poor prognosis and high mortality [2–6]. Therefore, finding new biomarkers that can early diagnose lung adenocarcinoma and evaluate the prognosis of lung adenocarcinoma patients is crucial to improve the survival rate of LUAD patients.

DARS2 has recently aroused the interest of researchers and has been found to be up-regulated in HCC [16], BLCA [13–15] and high-grade serous ovarian cancer [34]. Through bioinformatics and immunohistochemical staining, we found that the expression of DARS2 in LUAD samples was significantly higher than that in the control group. In vitro cell experiments have confirmed that interfering with the expression of DARS2 can significantly inhibit the proliferation and migration of LUAD cells, and can significantly promote tumor cell apoptosis. These results are consistent with previous research findings [17, 35, 36], further emphasizing the carcinogenic effect of DARS2 and its therapeutic potential in LUAD. At the same time, DARS2 has high specificity (98.4%) and sensitivity (95.2%) in the diagnosis of LUAD. Interestingly, in the latest research, Ucer et al. [37] found that DARS2 can be used as a new biomarker to distinguish malignant mesotheliom from LUAD. This result highlights the diagnostic value of abnormal expression of DARS2 in patients with LUAD.

In addition, this study is the first to explore the relationship between DARS2 expression and clinicopathological characteristics and ^18^F-FDG metabolic parameters in patients with LUAD. In this study, we found that the expression of DARS2 is related to the degree of differentiation, N stage, and TNM stage of LUAD patients in the clinical cohort, but not related to gender, age, and T stage. This may be due to heterogeneity [38, 39]. Jiang et al. analyzed the TCGA dataset and found that DARS2 was highly expressed in LUAD samples, and its expression level was related to tumor stage, T stage, and M stage. However, the relationship between DARS2 and clinicopathological characteristics was not verified by clinical cohort [35].

At the same time, this study also found that LUAD patients with high expression of DARS2 have a worse prognosis. In LUAD with high DARS2 expression, ^18^F-FDG PET/CT metabolic parameters were significantly higher than those in the group with low DARS2 expression, including SUVmax and SUVmean. Spearman correlation analysis also found a significant positive correlation between DARS2 expression and SUVmax, SUVmean, and TLG. Kishimoto et al. evaluated 169 patients with isolated lung cancer. Twenty-six patients had cancer recurrence, and SUVmax significantly increased compared to patients without recurrence. The disease-free survival rate was significantly reduced when SUVmax ≥ 2.5 compared to SUVmax < 2.5. Cox risk ratio model shows that SUVmax is an independent predictor of recurrence [40]. In a retrospective analysis of 55 patients with NSCLC, Lopci et al. found a statistically significant correlation between SUVmax, SUVmean, and stage and DFS. It was also found that there was a significant correlation between the metabolic parameters of FDG-PET and the expression of immune related markers in tumors [41]. ^18^F-FDG PET/CT is a commonly used medical imaging technique that uses radiolabeled ^18^F-deoxyglucose to detect metabolically active tissues and tumors in the body. In clinical practice, ^18^F-FDG PET/CT has been widely used in tumor diagnosis, staging, treatment response evaluation, and prognosis prediction [19, 20, 23, 40–42]. These findings further underscore the diagnostic and prognostic role of DARS2 as a newly discovered tumor marker in patients with LUAD. In addition, this study also found that SUVmax, a metabolic parameter of ^18^F-FDG PET/CT, is more effective in predicting DARS2 expression than SUVmean, TLG, and MTV. However, the molecular mechanism of how DARS2 enhances the glycolytic ability and ^18^F-FDG uptake of LUAD cells has not been reported.

Glycolysis is an important intracellular metabolic pathway that converts glucose into ATP for use by cells [43, 44]. In tumor cells, glycolytic pathways are often overactivated to provide sufficient energy and biosynthetic substances for tumor cells [18, 45]. Mitochondria are important organs responsible for energy metabolism in cells, and their normal function is crucial for the normal progression of glycolytic pathways [46]. DARS2 is a mitochondrial tRNA synthetase with defects associated with many neurological and mitochondrial diseases [7–9, 47]. Recent studies have shown that abnormal expression of DARS2 is also associated with the occurrence and development of various tumors [15, 16, 34, 35]. In this study, we found through GSEA that changes in DARS2 in the LUAD patient dataset resulted in a significant enrichment of glycolysis pathways in tumor cells. The expression of DARS2 is significantly correlated with glycolysis related genes. In vitro cell experiments have found that interfering with the expression of DARS2 can significantly inhibit the expression of glycolysis related genes (SLC2A1, GPI, ALDOA and PGAM1), thereby affecting the metabolism and proliferation of LUAD cells. Xia et al. found that downregulating the expression of SLC2A1 can inhibit the growth, migration, and invasion of LUAD cells [48]. Han et al. found that GPI was overexpressed in LUAD samples, and knocking down GPI could induce cell proliferation inhibition and cell cycle arrest [49]. Fu et al. found that ALDOA contributes to the activation of the EGFR/MAPK pathway, thereby promoting the expression of cyclin D1 and enhancing the proliferation of NSCLC [50]. Huang et al. have shown that allosteric inhibitor HKB99 can inhibit the expression of PGAM1, thereby inhibiting the growth and metastasis of NSCLC tumors [51]. These studies further suggest that DARS2 may promote the glycolysis process of LUAD cells by promoting the expression of SLC2A1, GPI, ALDOA, and PGAM1, thereby promoting the development of tumor cells. This study further demonstrates the important role of DARS2 in tumor metabolic pathways and its close relationship with glycolysis of tumor cells and ^18^F-FDG PET/CT imaging.

Although the role of DARS2 in tumor research needs further in-depth exploration and research, its important role in tumor metabolic pathways has received increasing attention. Future research can explore the potential application value of DARS2 in tumor therapy and molecular imaging of tumor cells. These findings will help to better understand the regulatory mechanisms of tumor metabolic pathways and bring more effective and personalized treatment strategies for cancer treatment.

## Conclusions

In summary, the results of this study indicate that DARS2 is highly expressed in LUAD tissue and is associated with tumor differentiation, N-stage, and TNM stage. At the same time, abnormal expression of DARS2 can accurately distinguish LUAD from normal tissue. Interfering with the expression of DARS2 can significantly inhibit the proliferation and migration of tumor cells, and further inhibit the glycolytic ability of LUAD cells by affecting the expression of key glycolytic genes. In addition, the expression level of DARS2 is closely related to the ^18^F-FDG PET/CT metabolic parameters of tumor cells. Therefore, DARS2 not only has potential value in the diagnosis and staging of LUAD, but also may become an important target for the treatment of LUAD. Treatment strategies aimed at DARS2 regulating mitochondrial function and glycolytic pathways can bring new possibilities for cancer treatment and provide more effective methods and means for clinical personalized treatment and imaging.

### Supplementary Information

**Additional file 1: Table S1.** The sequences of siRNAs used this study.

**Additional file 2: Table S2.** The sequences of primers used this study.

## Data Availability

The datasets generated during and/or analysed during the current study are available from the corresponding author on reasonable request.
